# National Consensus on Non-metastatic Castration-Resistant Prostate Cancer: more than just a snapshot

**DOI:** 10.1590/S1677-5538.IBJU.2020.0249.1

**Published:** 2021-02-03

**Authors:** Leonardo Oliveira Reis, Luciana Saboya Brito Dal Col, Marcus Vinícius Sadi

**Affiliations:** 1 Universidade Estadual de Campinas Campinas SP Brasil UroScience, Universidade Estadual de Campinas - Unicamp, Campinas, SP, Brasil; 2 Pontifícia Universidade Católica de Campinas Campinas SP Brasil Pontifícia Universidade Católica de Campinas - PUC-Campinas, Campinas, SP, Brasil; 3 Universidade Federal de São Paulo Escola Paulista de Medicina São Paulo SP Brasil Universidade Federal de São Paulo, Escola Paulista de Medicina – UNIFESP, São Paulo, SP, Brasil

## COMMENT

The landscape of castration-resistant prostate cancer (CRPC) has dramatically improved in the last few years. Still, it remains a very heterogeneous clinical setting. It ranges from patients with good performance status having an asymptomatic PSA elevation after hormone blockage failure with previous hormone-sensitive prostate cancer to those with a rapidly progressing disease and a dismal prognosis.

Non-metastatic castration-resistant prostate cancer (nmCRPC - M0) is a transient stage that affects almost 10% of prostate cancer patients, with up to 60% progressing to the metastatic disease within 5 years (1).

Recently 3 new androgen receptor blockers have been approved in Brazil by ANVISA for nmCRPC based on their level 1 pivotal studies – Spartan (apalutamide); Prosper (enzalutamide) and Aramis (darolutamide). These studies showed a remarkably similar result albeit targeting patients with slightly different characteristics (Table-1). Overall, the pooled analysis of the data revealed a significantly increased overall survival and improved progression-free survival due to these new agents compared with placebo.

**Table 1:** PROSPER vs. SPARTAN vs. ARAMIS.

Trial	PROSPER	SPARTAN	ARAMIS
Drug	Enzalutamide	Apalutamide	Darolutamide
Number of patients	1401	1207	1509
Drug (D) X Placebo (P)	933(D) vs. 468(P)	806(D) vs. 401(P)	955(D) vs. 554(P)
Median MFS in months (HR for metastasis or death; 95% CI, p-value)	36.6 vs. 14.7 (0.29; 0.24 – 0.35; p<0.001)	40.5 vs. 16.2 (0.28; 0.23 to 0.35; p<0.001)	40.4 vs. 18.4 (0.41; 0.34 – 0.50; p<0.001)
Median time to PSA progression in months (HR of PSA progression or death; 95% CI, p-value)	37.2 vs. 3.9 (0.07; 0.05 – 0.08, p<0.001)	NR vs. 3.7 (0.06; 0.05 – 0.08; p-value NR)	33.2 vs. 7.3 (0.13; 0.11 to 0.16; p<0.001)
Median overall survival in months (HR, 95% CI; p-value)	67 vs. 56.3 (0.73, 0.61 – 0.89; p = 0.0011) with median 48 months follow-up	73.9 vs. 59.9 (0.784, N/R, p = 0.0161) with median 52 months follow-up	NR vs. NR (0.69, 0.53 – 0.88; p = 0.003)
Median PSADT (D vs. P, months)	3.8 vs. 3.6	4.4 vs. 4.5	4.4 vs. 4.7
Any grade AE rate (D vs. P)	87% vs. 77%	96.5% vs. 93.2%	83.2% vs. 76.9%
Grade 3 or 4 AE rate (D vs. P)	31% vs. 23%	24.8% vs. 23.1%	24.7% vs. 19.5%
Grade 5 AE rate (D vs. P)	3% vs. 1%	1.2% vs. 0.3%	3.9% vs. 3.2%
Discontinuation rate (D vs. P)	9% vs. 6%	10.6% vs. 7.0%	8.9% vs. 8.7%

MFS = Metastasis-free survival; NR = Not reported; HR = Hazards ratio; CI = Confidence Interval; PSADT = PSA doubling time; AE = adverse effect.

In the current edition of the IBJU, Maluf et al. describe a national consensus of experts on nmCRPC, aiming to provide data on diverse topics such as diagnosis, patient selection, management of comorbidities, treatment efficacy, side effects due to the “inexistence of a national guideline for this clinical scenario” (2). It was not stated on the paper which criteria was used for selecting the Specialists nor which Medical Societies (if any) promoted the consensus; and, there was no disclaimer of how the consensus was supported.

The article brings a good review of the literature to hold up the expert's opinions. While every effort is expected from groups of experts to provide the best knowledge to diagnostic and treatments according to the most up-to-date data and international recommendations, one must not lose sight of the significant gaps and controversies that might coexist regarding clinically meaningful endpoints, financial toxicity, overtreatment, pharmacoeconomic and polypharmacy for this type of cancer patients, especially in a country as large and heterogeneous as Brazil, which may provide all available resources in one area but may lack significant basic means in several others.

Thus, several important points deserve consideration.

The consensus is somehow outdated; although the authors do mention data from the Aramis study, darolutamide was not included in the experts' questions and responses because it was only approved in Brazil by December 2019.

The panel agreed to answer questions on the assumption of the existence of an ideal clinical scenario based on the best evidence available. Yet it was unable to reach consensus in any of the questions related to staging tools, giving no guideline to the average urologist. This is of utmost importance considering that PET-PSMA is currently available in some major cities throughout the country and that the patients included in the pivotal studies had greater than a 95% chance of having nodal or bone metastasis had they been subjected to a staging PET-PSMA. New data suggests that these patients are all under-staged and should be considered to have low-volume metastatic disease rather than nmCRPC (1).

In this regard, one could question the responses regarding whether a drug that has been approved for both nmCRPC and M1-CRPC such as enzalutamide would not be the most obvious choice in a perfect scenario. On the other hand, in an indirect comparison made by 2 independent groups (3, 4), apalutamide and enzalutamide were significantly more effective than darolutamide concerning metastasis-free survival and PSA progression-free survival; darolutamide, however, showed potential for a better-tolerated drug.

These data are in line with preclinical studies in which apalutamide shows a higher therapeutic index and a greater opportunity for dose escalation. Additionally, apalutamide is molecularly and mechanistically similar to enzalutamide, both CYP-inhibitors with potential CYP mediated drug––drug interactions, while darolutamide, though not free from interactions and adverse effects, has a distinct molecular structure with low blood-brain barrier penetration and no CYP-inhibition (5).

Another important point relates to PSA doubling time (PSADT); the actual median PSADT in the pivotal studies was less than 5 months so the consideration for introducing these new drugs demands the evidence of rapid disease progression, in which a positive PSMA PET-PSMA imaging might be the rule, not supporting treatment for all nmCRPC patients.

However, like the US Food and Drug Administration (FDA), the ANVISA labels for enzalutamide, apalutamide, and darolutamide do not specify PSADT as a clinical criterion, which could favor extrapolation of data and unscrupulous use of these drugs. On the other hand, the European Medicines Agency (EMA) labels do specify the less than < 10 months PSADT used in the trials for selecting patients (3).

Physicians treating nmCRPC face challenges in treatment decisions. Consensus such as this may provide a useful mechanism to synthesize available evidence together with expert opinion. However, the data may only reflect a snapshot in a rapid moving area; besides, the agreement among experts does not mean surety, because experts can be influenced by data, individual experiences, momentum, practice setting, the peers' sentiments, financial conflicts of interest, trials they conducted, among other scenarios (6).

As such, care must be taken when analyzing responses so as not to harm patients which may suffer both from disease progression because they were not exposed to new and better treatments in a timely fashion but also by inadequate use of drugs. The pivotal studies demonstrate that as a class effect, there are higher rates of fatigue, falls, fractures and cardiovascular events, even death, with the use of the new generation of antiandrogens when compared to placebo (7).

Given the lack of a head-to-head study among these new drugs so far, a network meta-nalysis would offer a methodologically stronger strategy to facilitate individualized treatment selection, indirectly comparing the safety and efficacy of these therapies. However, even such meta-nalysis should be considered with caution because patients' characteristics may be significantly different between the trials.

For example, there was no minimum serum PSA and intriguingly higher adverse effect on the placebo group in the SPARTAN trial compared to ARAMIS and PROSPER that used 2 ng/ml minimum PSA. The PROSPER trial included only patients without lymph node enlargement (N0) by CT/MRI while the SPARTAN and ARAMIS trials included patients with lymph nodes up to 2 cm (N1) below aortic bifurcation. The importance of these details for drug selection remains to be assessed.

Most responses in the consensus were aligned to international guidelines such as the extensively cited NCCN guidelines version 1.2020 or the APCCC 2019 consensus (8). Taking into consideration that we live in a globalized world and considering the existence of several guidelines and reviews from recognized international societies and experts summarizing the best evidence available, one might question if a national consensus where experts evaluate only “an ideal scenario based on the best clinical evidence” is in fact, ideal, and if other landscapes should also have been explored. One could wish for a more real-life analysis, which might have included other acceptable alternatives if any still exists for such a clinical scenario, under our pharmacoeconomic perspective and considering our highly variable socioeconomic structure. While darolutamide, apalutamide, and enzalutamide are already ANVISA approved, it does not mean they are available to the majority of the Brazilian population.

While a national consensus might help non-specialists in when and how to choose among the new antiandrogens, experts usually consult major international guidelines, and most aspects explored in this consensus were somehow widely tackled in the APCCC 2019 (8), and also are available at the NCC guidelines, which might drive readers to consume these more comprehensive documents in detriment of this one (2).

We all share responsibility for ensuring that medical practice is driven by up-to-date, strong, unbiased evidence, and the relatively long overall survival period of nmCRPC now clearly apparent, warrants attention regarding the future care of these patients, puzzling where to set the bar.
